# Isolated Hand Palsy in National Institutes of Health Stroke Scale (NIHSS): Is It Useful?

**DOI:** 10.5811/westjem.2018.2.37654

**Published:** 2018-04-05

**Authors:** Antonio Siniscalchi, Piergiorgio Lochner, Paolo Perrotta, Stefano Rizzuto, Giovambattista De Sarro, Luca Gallelli

**Affiliations:** * Annunziata Hospital, Department of Neurology, Cosenza, Italy; † Saarland University Medical Center, Department of Neurology, Hamburg, Germany; ‡ University of Piedmont East A. Avogadro, Department of Translational Medicine, Section of Neurology, Novara, Italy; § Annunziata Hospital, Department of Neuroradiology, Cosenza, Italy; ¶ University of Catanzaro Clinical Pharmacology, School of Medicine, Department of Health Science, Catanzaro, Italy; || Mater Domini University Hospital, Pharmacovigilance Unit, Catanzaro, Italy

Stroke is the third cause of death in industrialized countries,^1^ and the main cause of neurological disability in the world.^1^ This has led to the need to significantly evaluate the effectiveness of an intervention both at the individual patient level as well as in clinical trials. The meaning of stroke-survivor recovery should be described by more sophisticated measures than are required by simple dichotomous end points, i.e., mortality or stroke recurrence.

The National Institutes of Health Stroke Scale (NIHSS) is a 42-point impairment scale that could be administered reliably by a variety of clinicians in a relatively short time to reveal neurological symptoms and signs in patients with stroke in a standardized manner.^2^,^3^,^4^ The NIHSS provides an ordinal, nonlinear measure of acute stroke-related impairments by assigning numerical values to various aspects of neurological function.^2^,^3^ This scale is used to quickly evaluate the stroke severity before and after each treatment.^2^,^3^,^4^ The NIHSS takes about six minutes to perform, with no need for additional equipment, and is easily executable after training in how to use it.^3^,^5^

NIHSS scores are reliable across observers, as shown when used both by neurology-trained and non-neurologist raters. The potential to perform a neurological exam with a reliable method suitable for nonspecialists is an important strength of the NIHSS. NIHSS reliability and validity has also been demonstrated for remote assessment via telemedicine.^2^ The NIHSS also has predictive validity. In fact, the initial NIHSS score is a robust predictor of in-hospital complication and outcome at three months.^3^,^4^,^5^ The validity of NIHSS derives from correlations with objective measures of stroke severity, such as size of infarct on imaging. 3,5

Developed in the early 1980s, the NIHSS is used in acute-stroke studies, particularly in early trials regarding thrombolytic and neuroprotectant treatments.^5^ The NIHSS was developed through a robust consensus approach, taking the most informative measures from existent stroke-examination scales (Toronto Stroke Scale, Oxbury Initial Severity Scale, and Cincinnati Stroke Scale) and creating a composite scale that was further reviewed by a panel of stroke researchers and then amended. Further items were added to ensure the assessment was as comprehensive as possible. It has been used as a primary endpoint during trials with thrombolytic agents and patients with acute stroke.

The NIHSS is composed of the following elements: level of consciousness, horizontal eye movements, visual field test, facial palsy, motor arm, motor leg, limb ataxia, sensory abilities, language, dysarthria, and extinction and inattention (attention to surrounding environment). The score extends from 0 (normal neurological examination) to 42 (unresponsiveness coma). A score of 10 or higher is more probably related to a large-artery occlusion.^2^,^3^ The score has a good correlation with anterior circulation stroke but underestimates clinical severity in posterior circulation stroke.^5^ It is well recognized that while an individual can score 0 on the NIHSS, he might have an ischemic stroke, in particular in the posterior circulation area.^3^

Although some items related to the anterior-circulation infarction in NIHSS can be scored, other elements such as isolated hand/fingers palsy receive no score. Isolated hand/fingers palsy is not included in the NIHSS because it is a rare type of stroke. This non-inclusion could represent a weakness of the NIHSS because this type of palsy is a disabling clinical condition. Because the NIHSS is an impairment scale, it can provide only limited information about individual stroke survival. For example, an NIHSS score of 1 is considered an “excellent” outcome from stroke; a hemianopia would score NIHSS 1, but for the individual this may not seem an “excellent” result because this symptom precludes driving and may cause loss of employment. However, isolated hand palsy in patients with cortical lesions has been rarely reported,^6^ probably because this isolated nerve palsy is misdiagnosed as peripheral nerve lesions. The incidence of acute stroke with isolated hand paresis is not known, but it seems to constitute between 0.83% to 1.5 % of all ischemic strokes.^7^

Previous studies have shown that a paralysis of hand and fingers without sensory deficit is due to the cortical infarction of the precentral dial knob.^6^ Anatomically, the precentral knob is a knob-like segment of the precentral gyrus projecting to the middle genu of the central sulcus, which is known as a reliable landmark for the motor hand area.^8^ Moreover, the medial and lateral portions of the precentral knob are responsible for the ulnar and radial side of the fingers,^6^,^8^ and it is consistent with the classical description of motor somatotopy in Penfield’s homunculus.^9^ Embolic mechanisms were more often associated with small cortical infarction associated with isolated hand/fingers palsy.^6^ Clinicians must consider that patients with isolated hand palsy may have an alternative explanation, including a history of pain suggesting vasculitis, waking from sleep with the deficit (compression), fall (trauma), and shoulder (neuralgic amyotrophy) or neck pain with radicular symptoms. In the absence of these findings, the patient should be aggressively treated for acute ischemic stroke.

An ischemic stroke in patients without large cerebral-vessel occlusion or peripheral occlusions on intra-arterial digital subtraction arteriography have a low NIHSS score and a favorable outcome to thrombolytic treatment.^10^ However, the use of diffusion-weighted magnetic resonance imaging could help clinicians diagnose ischemic stroke, including cerebral small ischemic lesions.^4^ The delayed diagnosis of acute stroke or absence of thrombolytic therapy may induce the involvement of corticofugal tracts on arm and hand recovery with consequent disability.^11^ One might question, however, whether the absence of this item for isolated hand palsy in NIHSS might underestimate the effect of thrombolytic therapy. There are no data about the benefit of thrombolytic therapy in this type of stroke, even though it has been reported that intravenous thrombolytic therapy is more effective in embolic stroke (i.e., isolated hand/fingers palsy) compared to atherothrombotic stroke.^11^

Hence, we suggest the inclusion of this finding to improve the sensitivity of NIHSS for anterior circulation stroke. If the NIHSS were to be expanded by adding this item for isolated hand palsy, it could have an impact on clinical and therapeutic trials, as well as on outcomes of anterior strokes. This could also be useful to maintain a high index of suspicion for acute stroke, particularly of anterior circulation, in the presence of NIHSS score = 0. We suggest adding one category to the existing item 7 of the NIHSS (Table). When neurological examination, particularly in patients with vascular risk factors, reveals an acute, isolated hand palsy without sensory deficit, we can use this modified NIHSS to diagnose a cortical brain lesion.

In conclusion, patients with isolated hand palsy do not receive an evaluation score. Thus, further studies are needed to consider this important central sign in the current NIHSS score, and open the possibility of adequate and aggressive treatment.

## Figures and Tables

**Table:** National Institute of Health Stroke Scale (NIHSS) item 7 (current) and suggested modification to item 7.

Item 7, NIHSS current	Item 7, NIHSS modified
0 = No drift; limb holds 90 (or 45) degrees for full 10 seconds	0 = No drift; limb holds 90 (or 45) degrees for full 10 seconds
1 = Drift; limb holds 90 (or 45) degrees, but drifts down before full 10 seconds; does not hit bed or other support.	1 = Drift; limb holds 90 (or 45) degrees, but drifts down before full 10 seconds; does not hit bed or other support.
2 = Some effort against gravity; limb cannot get to or maintain (if cued) 90 (or 45) degrees, drifts down to bed, but has some effort against gravity.	2 = Some effort against gravity; limb cannot get to or maintain (if cued) 90 (or 45) degrees, drifts down to bed, but has some effort against gravity.
3 = No effort against gravity; limb falls.	3 = No effort against gravity; limb falls.
	3= isolated hand palsy
4 = No movement.	4 = No movement.

## References

[1] Siniscalchi A, Iannacchero R, Anticoli S (2016). Anti-inflammatory strategies in stroke: a potential therapeutic target. Curr Vasc Pharmacol.

[2] Pezzella FR, Pozzessere C, Siniscalchi A (2013). The cloud stroke unit: 24-hour acute stroke expertise-on-demand. Hosp Top.

[3] Siniscalchi A, Sztajzel R, Malferrari G (2017). The National Institutes of Health Stroke Scale: its role in patients with posterior circulation stroke. Hosp Top.

[4] Anticoli S, Bravi MC, Perillo G (2016). Effect of cardioembolic etiology on intravenous thrombolysis efficacy for acute ischemic stroke. Curr Neurovasc Res.

[5] Harrison JK1, McArthur KS, Quinn TJ (2013). Assessment scales in stroke: clinimetric and clinical considerations. Clin Interv Aging.

[6] Kim JS (2001). Predominant involvement of a particular group of fingers due to small, cortical infarction. Neurology.

[7] Alstadhaug KB, Sjulstad A (2013). Isolated hand paresis: a case series. Cerebrovasc Dis Extra.

[8] Yousry TA, Schmid UD, Alkadhi H (1997). Localization of the motor hand area to a knob on the precentral gyrus. Brain.

[9] Schott GD (1993). Penfield’s homunculus: a note on cerebral cartography. J Neurol Neurosurg Psychiatry.

[10] Arnold M, Nedeltchev K, Brekenfeld C (2004). Outcome of acute stroke patients without visible occlusion on early arteriography. Stroke.

[11] Nam HU, Huh JS, Yoo JN (2014). Effect of dominant hand paralysis on quality of life in patients with subacute stroke. Ann Rehabil Med.

